# Growth-arrest-specific 7C protein inhibits tumor metastasis via the N-WASP/FAK/F-actin and hnRNP U/β-TrCP/β-catenin pathways in lung cancer

**DOI:** 10.18632/oncotarget.6229

**Published:** 2015-10-25

**Authors:** Ruo-Chia Tseng, Jer-Wei Chang, Jiou-Shan Mao, Charng-Dar Tsai, Pei-Chen Wu, Cuei-Jyuan Lin, Yi-Lin Lu, Sheng-You Liao, Hung-Chi Cheng, Han-Shui Hsu, Yi-Ching Wang

**Affiliations:** ^1^ Department of Molecular Biology and Human Genetics, College of Life Science, Tzu Chi University, Hualien, Taiwan; ^2^ Institute of Medical Sciences, Tzu Chi University, Hualien, Taiwan; ^3^ Institute of Molecular and Genomic Medicine, National Health Research Institutes, Zhunan, Taiwan; ^4^ Department of Pharmacology, College of Medicine, National Cheng Kung University, Tainan, Taiwan; ^5^ Department of Life Sciences, National Taiwan Normal University, Taipei, Taiwan; ^6^ Institute of Basic Medical Sciences, College of Medicine, National Cheng Kung University, Tainan, Taiwan; ^7^ Department of Biochemistry and Molecular Biology, College of Medicine, National Cheng Kung University, Tainan, Taiwan; ^8^ Division of Thoracic Surgery, Taipei Veterans General Hospital, Institute of Emergency and Critical Care Medicine, National Yang-Ming University School of Medicine, Taipei, Taiwan

**Keywords:** GAS7C, N-WASP, hnRNP U, lung cancer, prognosis

## Abstract

Growth-arrest-specific 7 (GAS7) belongs to a group of adaptor proteins that coordinate the actin cytoskeleton. Among human GAS7 isoforms, only GAS7C possesses a Src homology 3 domain. We report here that GAS7C acts as a migration suppressor and can serve as a prognostic biomarker in lung cancer. GAS7C overexpression reduces lung cancer migration, whereas GAS7C knockdown enhances cancer cell migration. Importantly, ectopically overexpressed GAS7C binds tightly with N-WASP thus inactivates the fibronectin/integrin/FAK pathway, which in turn leads to the suppression of F-actin dynamics. In addition, overexpression of GAS7C sequesters hnRNP U and thus decreases the level of β-catenin protein via the β-TrCP ubiquitin-degradation pathway. The anti-metastatic effect of GAS7C overexpression was also confirmed using lung cancer xenografts. Our clinical data indicated that 23.6% (25/106) of lung cancer patients showed low expression of *GAS7C* mRNA which correlated with a poorer overall survival. In addition, low *GAS7C* mRNA expression was detected in 60.0% of metastatic lung cancer patients, indicating an association between low *GAS7C* expression and cancer progression. A significant inverse correlation between mRNA expression and promoter hypermethylation was also found, which suggests that the low level of *GAS7C* expression was partly due to promoter hypermethylation. Our results provide novel evidence that low GAS7C correlates with poor prognosis and promotes metastasis in lung cancer. Low GAS7C increases cancer cell motility by promoting N-WASP/FAK/F-actin cytoskeleton dynamics. It also enhances β-catenin stability via hnRNP U/β-TrCP complex formation. Therefore, GAS7C acts as a metastasis suppressor in lung cancer.

## INTRODUCTION

Despite significant improvements in both diagnostic and therapeutic modalities in lung cancer treatment, outcomes remain poor when the disease has spread to the regional lymphatics [1]. In this context, identification of the genes and molecular pathways involved in lung cancer metastasis opens up the possibility of advances in lung cancer therapeutics. Our previous studies have shown that there is a high frequency of loss of heterozygosity at 17p13.1 to 17p13.2, which includes the chromosomal site of the *growth-arrest-specific 7* (*GAS7*) gene. This loss of heterozygosity has been found to be associated with a decrease in *GAS7* transcription in lung cancer patients, indicating a potential role for GAS7 as a tumor suppressor in lung cancer [2, 3].

Gas7 was first isolated by expression of a chromosomally-inserted retrovirus-based *lacZ* reporter gene following serum starvation of mouse NIH3T3 cells [4]. The *GAS7* gene is transcribed as three isoforms, GAS7A, GAS7B and GAS7C, through alternative splicing [5, 6]. The GAS7 protein consists of a series of different functional domains: Src homology 3 domain (SH3), WW domain, and FES-CIP4 homology (FCH) domain from the N-terminal to the C-terminal. Among these functional domains, the SH3 domain is present only in the GAS7C isoform [7]. SH3 domains are able to bind to proline-rich ligands and change the subcellular localization of the bound protein. SH3 domains are found in proteins associated with signaling pathways that regulate the cytoskeleton, such as the Ras and Src proteins [8].

Ingham and associates used tandem mass spectrometry (MS) to identify human polypeptides that associate with ten human WW domains and these included GAS7 [9]. They identified several GAS7 WW domain-associated proteins including Neural-Wiskott Aldrich syndrome protein (N-WASP), a key regulator of actin dynamics, and heterogeneous nuclear ribonucleoprotein U (hnRNP U), a pseudosubstrate of the β-TrCP E3 ubiquitin ligase complex. Later, You *et al*. found that GAS7 physically interacts with N-WASP through the WW domain and this may lead to the formation of membrane protrusions via recruitment of the Arp 2/3 complex to increase the aberrant neurite outgrowth of hippocampal neurons in a mouse model [10]. The WW domain is architecturally similar to the SH3 domain [11]. Since human GAS7C contains both SH3 domain and WW domains, it has been speculated that human GAS7C may function similarly to mouse Gas7 in N-WASP activating cytoskeleton rearrangement and microfilament rearrangement [10], which correlate with cell migration [12, 13]. It is also known that activation of the fibronectin/integrin/focal adhesion kinase (FAK)/N-WASP pathway promotes cell migration [14]. The activation of integrin receptors recruits FAK signaling proteins, as well as actin dynamic associated proteins such as N-WASP and Arp 2 inducing microfilament rearrangement and cell migration. However, the relationship between GAS7C and the fibronectin/integrin/FAK pathway have never been demonstrated in human cancer.

Like many other hnRNPs, hnRNP U can shuttle in and out of the nucleus, yet reside predominantly in the nucleus. hnRNP U is also a pseudosubstrate of the β-TrCP E3 ubiquitin ligase complex [15, 16]. hnRNP U dissociates from the β-TrCP complex in the presence of a true substrate protein such as p-β-catenin [17]. The β-TrCP complex then binds to p-β-catenin, which is subject to degradation through the ubiquitin-proteasome pathway; this results in the inhibition of cell migration [18]. Decreased expression of the β-catenin-degradation complex has been shown to lead to β-catenin nuclear accumulation in lung cancer patients [19]. Although hnRNP U associates with the WW domain of GAS7, little is known about the effect and the mechanism of this interaction between GAS7 and hnRNP U in cancer cells.

To date, the clinical and biological significance of GAS7C has never been demonstrated in human cancer patients. To investigate the roles of GAS7C in tumor progression, we performed a series of molecular analyses of the activity of the N-WASP/FAK/F-actin pathway proteins and the formation of hnRNP U/β-TrCP/β-catenin complex in relation to the expression of GAS7C using the lung cancer model. The clinical link between these proteins and the mechanism of anti-migratory effects of GAS7C during lung cancer progression were further explored.

## RESULTS

### GAS7C is the major alterative isoform in human lung cancer cell lines

**Figure 1:**
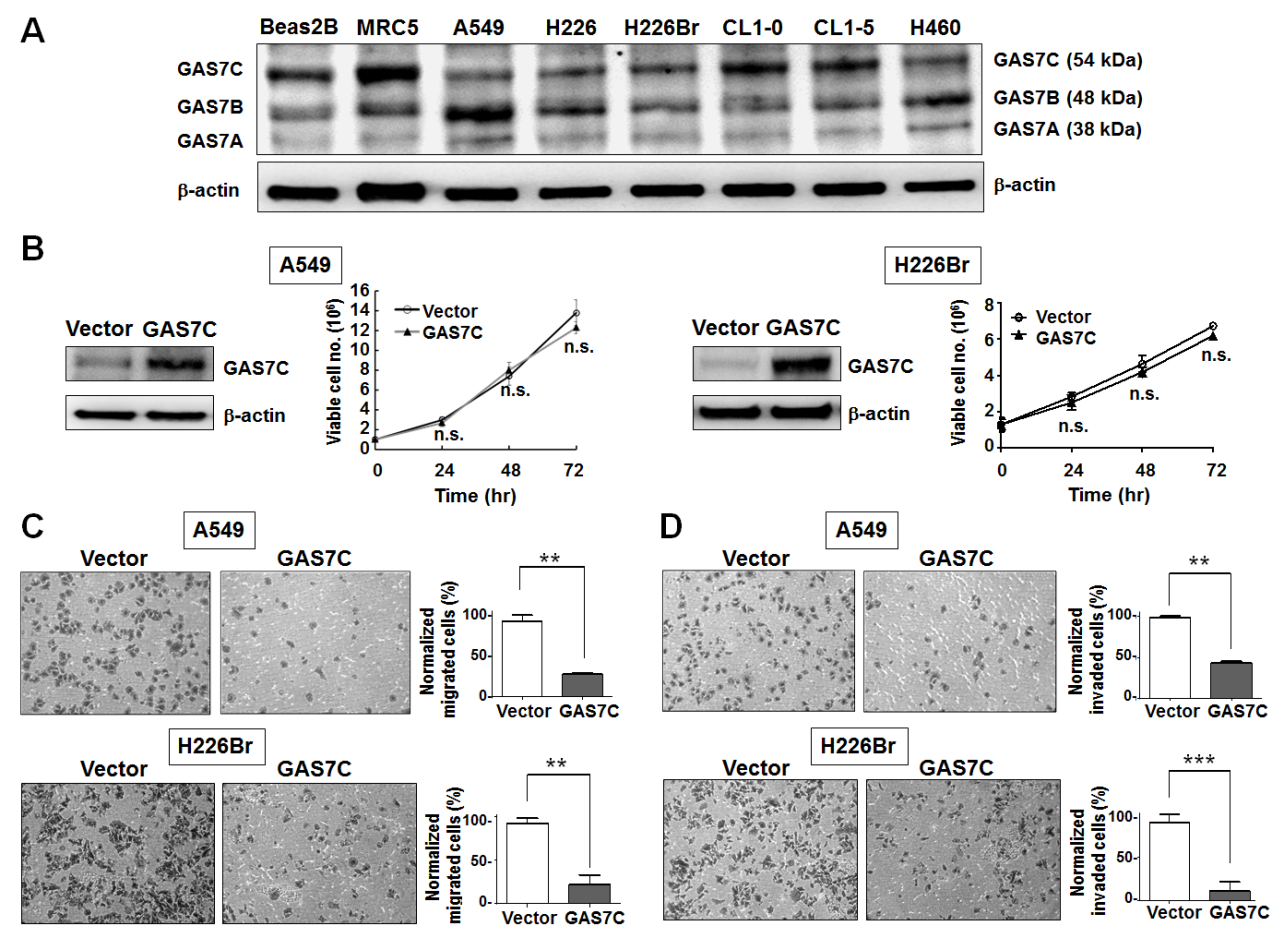
GAS7C overexpression inhibits cell migration ability in lung cancer cells **A.** Western blot analyses of expression of GAS7 isoforms in a panel of two normal lung cell lines (Beas2B and MRC5) and six human lung cancer cell lines (A549, H226, H226Br, CL1-0, CL1-5, and H460). β-actin levels are shown as a loading control for the total protein lysate. **B.** GAS7C overexpression did not affect cell proliferation of A549 and H226Br cells within 72 hr post-transfection. The expression of ectopic GAS7C was confirmed by Western blotting (left). **C.** Transwell-migration assays showed that GAS7C overexpression decreased cell migration ability. **D.** Transwell-invasion assays showed that GAS7C overexpression decreased cell invasion ability. Quantitative data are presented as the mean ± SD from three independent experiments. n.s., non-significant; **p < 0.01; ***p < 0.001.

Since the tissue distribution of GAS7C has never been reported, we first examined the mRNA expression of *GAS7C* using isoform-specific PCR in commercialized human tissue cDNA panels. The results revealed that GAS7C expression was high in lung and liver tissues, but that expression was low in other tissues (Supplementary Fig. 1). To examine the protein expression of GAS7 isoforms in the lung cell model, we performed Western blot analysis of protein extracts from two human normal lung cell lines (Beas2B and MRC5) and six lung cancer cell lines (A549, H226, H226Br, CL1-0, CL1-5, and H460). The expression levels of the GAS7A, GAS7B and GAS7C proteins varied among these cell types (Fig. 1A). GAS7A protein expression was barely detectable in all cells examined, including the normal lung cell lines. Both GAS7B and GAS7C were expressed in Beas2B and MRC5 normal lung cells. The GAS7B protein was expressed at a similar level in both normal and cancer cells. Importantly, the level of GAS7C protein expression was relatively lower in most of the lung cancer cell lines examined, namely A549, H226, H226Br, and H460, than that in the normal cell lines. These results suggest that GAS7C is the major alterative isoform of this protein in lung cancer cell lines.

### GAS7C overexpression decreases lung cancer cell migration and invasion

**Figure 2:**
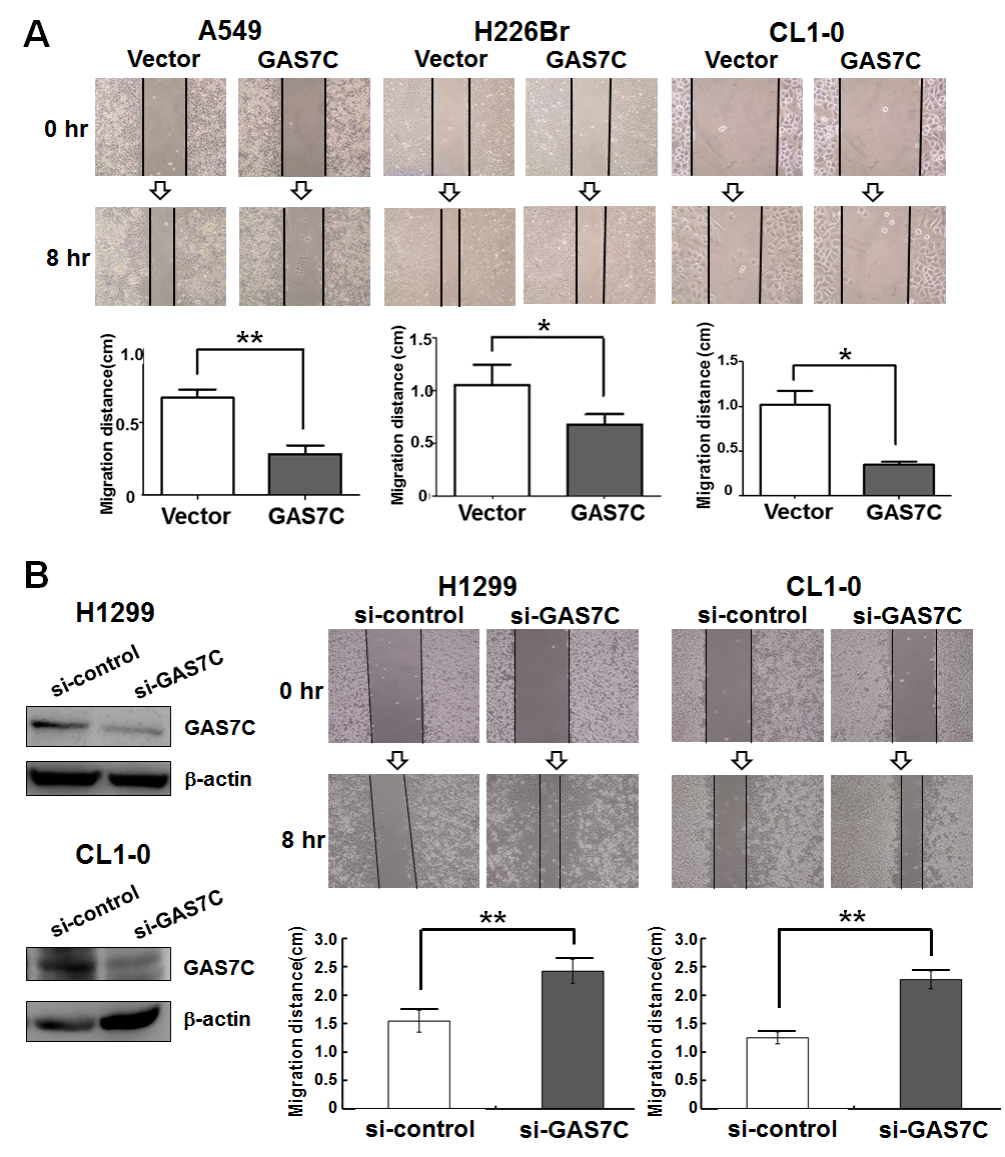
GAS7C overexpression decreases cell motility as assessed by wound healing assay Cells in culture were monitored for their ability to migrate into the created wound gap. The created wound gap was photographed at 0 and 8 hr. **A.** The wound healing images demonstrated that A549, H226Br and CL1-0 cells overexpressing GAS7C migrated much slower than vector control cells. Quantitative wound healing data of the various lung cancer cell lines are shown in lower panel. **B.** Knockdown of GAS7C increased lung cancer cell migration ability. Lung cancer cells were transiently transfected with si-control or si-GAS7C oligos. The protein expression level of the manipulated GAS7C is shown in left panel and quantitative data are shown in lower panel. Quantitative data are present as the mean ± SD from three independent experiments. *p <0.05; **p <0.01.

As previously discussed, GAS7C contains both SH3 and WW domains which bind to many proteins involved in microfilament rearrangement and cell migration. To examine whether GAS7C plays a regulatory role in cell motility control, we carried out wound healing, transwell-migration and transwell-invasion assays using various lung cancer cells that were ectopically expressing GAS7C. It should be noted that the proliferation rate of these GAS7C overexpression cells did not differ from that of the vector control cells during the time allowed for migration or invasion (Fig. 1B). Using the transwell-migration assay, the number of GAS7C overexpressing A549 and H226Br cells that had migrated through the porous membrane was significantly fewer than those of the vector control (Fig. 1C). In addition, we observed that GAS7C overexpression cells invaded through the Matrigel at a lower level compared to the vector control cells using a transwell-invasion assay (Fig. 1D). Using the wound healing assay, it was found that GAS7C overexpression cells migrated at a significantly much slower rate at the leading edge of the scratch wound compared with the vector control cells for all cell lines examined, namely the A549, H226Br, and CL1-0 cell lines (Fig. 2A). To further confirm the inverse correlation between GAS7C expression and cell motility in lung cancer, we used siRNA to generate knockdown of GAS7C in the A549, CL1-0, and H1299 lung cancer cell lines. GAS7C knockdown cells then had their cell motility assessed using the wound-healing assay (Fig. 2B) and the transwell assay (Supplementary Fig. 2). The results indicated that GAS7C knockdown cells showed a significantly increased migratory capacity compared with the si-control cells. Together, these results indicated that GAS7C overexpression dramatically reduced cell migration and invasion capacity using a variety of lung cancer cell models.

### GAS7C overexpression decreases lung metastases in an experimental metastasis animal model

**Figure 3:**
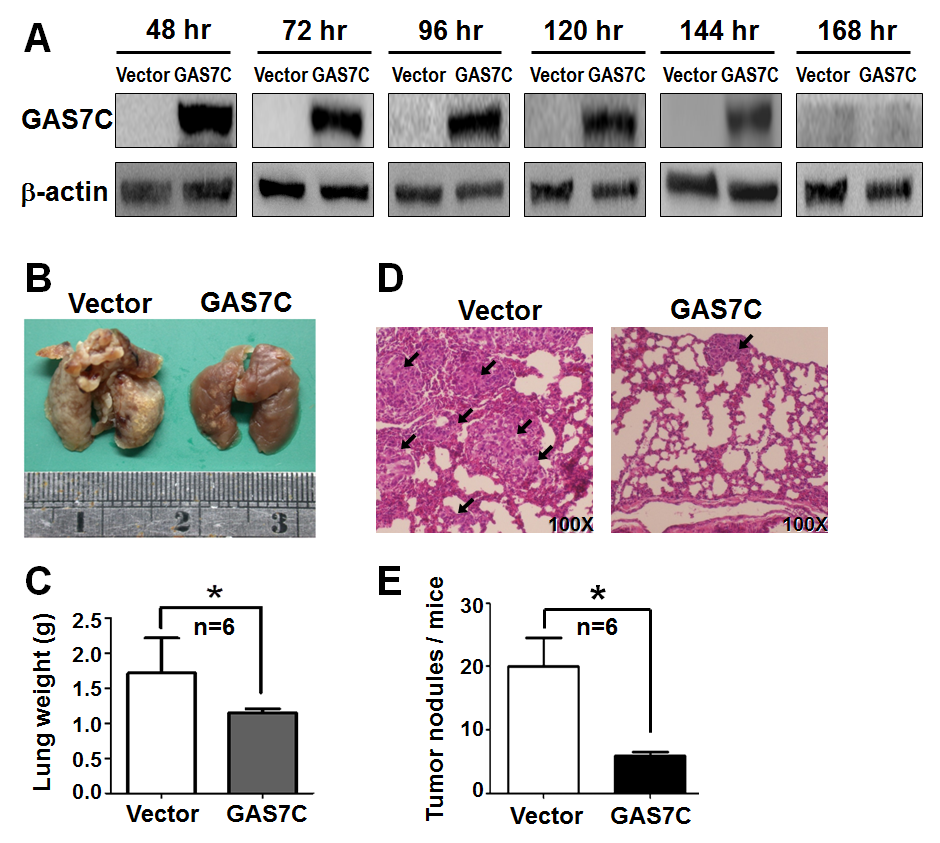
GAS7C overexpression reduces lung metastases of A549 cancer cells in animals **A.** The amount of GAS7C protein in A549 cells present was monitored by Western blotting from 48 hr to 168 hr post-transfection. **B.** Representative lung images taken from mice after five weeks are shown. The group of animals injected with A549 cells overexpressing GAS7C showed significantly fewer tumor nodules in their lung tissue than vector control group. **C.** Quantitative data showing the lung weight indicates that the size of the lung tissue was smaller for the GAS7C overexpression group than the vector control group. **D.** H&E staining of tumor metastasis is shown. Tumor nodules are indicated by arrows (Original magnification: 100X). **E.** Quantitative data on the tumor nodules present in the various lung samples are shown. Data are presented as the mean ± SD (*n* = 6 mice per group). *p < 0.05.

We then carried out an *in vivo* experimental metastasis assay to further characterize the effect of GAS7C overexpression on lung cancer metastasis. GAS7C overexpression or control A549 cells were injected via the tail vein into six nude mice per experimental group. Initially, we monitored GAS7C protein expression by Western blotting of cell lysates obtained at various time from 48 hours to 168 hours post-transfection. The results indicated that GAS7C was continuously expressed up to 144 hours using a cell-based assay, which allowed sufficient time for the GAS7C overexpression cells to reach and colonize in the lung tissue via the blood circulation (Fig. 3A). After five-weeks, the animals were sacrificed for lung tissue examination. The number of tumor nodules on the surface of lungs from GAS7C overexpression group was significantly fewer than the vector control group (Fig. 3B). In addition, the GAS7C overexpression group was found to have a significantly lighter lung tissue weight than the control group (Fig. 3C). H&E staining confirmed that the tumors from the GAS7C overexpression group had a smaller mass and a well-differentiated phenotype. However, the tumors from the control group had a greater tumor mass and poorly differentiated phenotype (Fig. 3D). In addition, mice group of GAS7C overexpression showed less tumor nodules in lung than did vector control mice (Fig. 3E). These *in vivo* results corroborate our *in vitro* data supporting the hypothesis that overexpression of GAS7C suppresses tumor metastasis.

### GAS7C binds with N-WASP to inhibit the fibronectin/integrin/FAK pathway and actin arrangement

**Figure 4:**
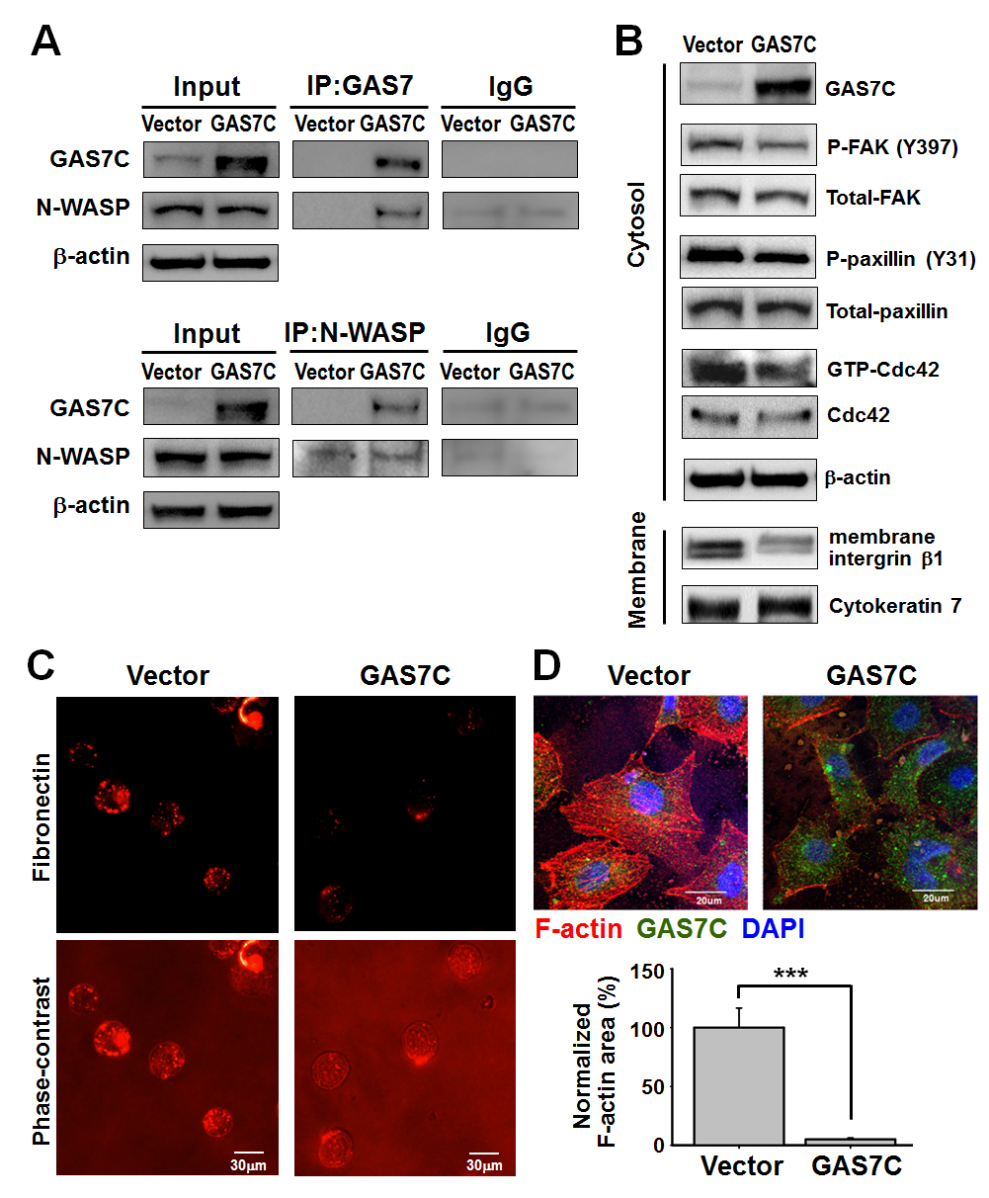
GAS7C protein is associated with N-WASP, which is involved in the fibronectin/integrin/FAK/F-actin dynamics pathway **A.** Protein lysates of A549 cells expressing control (vector) or GAS7C (GAS7C) were immunoprecipitated (IP) using anti-GAS7 (upper) or anti-N-WASP (lower) antibody. The IP proteins were analyzed by Western blotting using the indicated antibodies. Normal IgG served as negative control. More N-WASP was associated with GAS7C in the GAS7C overexpressing cells than in the vector control cells. **B.** Western blot analysis of actin dynamics-related proteins in vector and GAS7C overexpressing A549 cells. Decreased P-FAK (Y925), P-paxillin (Y31), and membrane integrin β1 proteins level, along with a reduced Cdc42 activity, were observed in GAS7C overexpression cells. **C.** Reduction of membrane fibronectin staining (red fluorescent) in GAS7C overexpression cells compared with the vector control cells was found by fluorescent microscopy. Scale bar: 30 μm. **D.** A reduction in F-actin staining was observed in GAS7C overexpression cells compared with the vector control cells. The F-actin was analyzed by phalloidin staining (red fluorescent). GAS7 (green) and nucleus staining (blue) are also shown. Scale bar: 20 μm. Quantitative data obtained from the stained tissue samples are shown.

We further studied the molecular mechanism that underlies the suppression of migration and invasion by GAS7C. N-WASP, a key regulator in actin dynamics [20], has been previously suggested as a GAS7 WW domain association protein by proteomic screening [9]. We performed immunoprecipitation (IP)-Western blot assay to confirm the association between N-WASP protein and GAS7C in A549 cells ectopically expressing GAS7C or vector control. As shown in Fig. 4A, overexpression of GAS7C increased the association between GAS7C and N-WASP as evidenced by the results of the IP and reverse IP-Western blot assays. It is known that activation of the fibronectin/integrin/FAK signaling pathway plays a promoting role in actin rearrangement and cell motility [14]. Our Western blot results showed that GAS7C overexpression decreased the levels of fibronectin/integrin/FAK pathway proteins such as p-FAK (pY925), p-paxillin (pY31) and membrane integrin β1. Cdc42 activity was also reduced when GAS7C was overexpressed (Fig. 4B). Fibronectin expression enhances tumor cell motility, cancer spread, and metastasis [21, 22]. Therefore, we carried out immunocytochemisty staining to examine the level of fibronectin on plasma membrane of suspended cells. The results indicated that GAS7C overexpression dramatically reduced the level of fibronectin present on the cell membrane compared with the vector control cells (Fig. 4C). Furthermore, immunofluorescent staining also showed that there was less intense F-actin staining in the GAS7C overexpression cells compared to the vector control cells (Fig. 4D). Taken together, these results suggested that GAS7C is able to interact with N-WASP thereby decreasing the activity of the fibronectin/integrin/FAK pathway proteins and altering the level of actin arrangement.

### GAS7C sequesters hnRNP U to stimulate β-catenin degradation by the β-TrCP ubiquitin ligase complex

**Figure 5:**
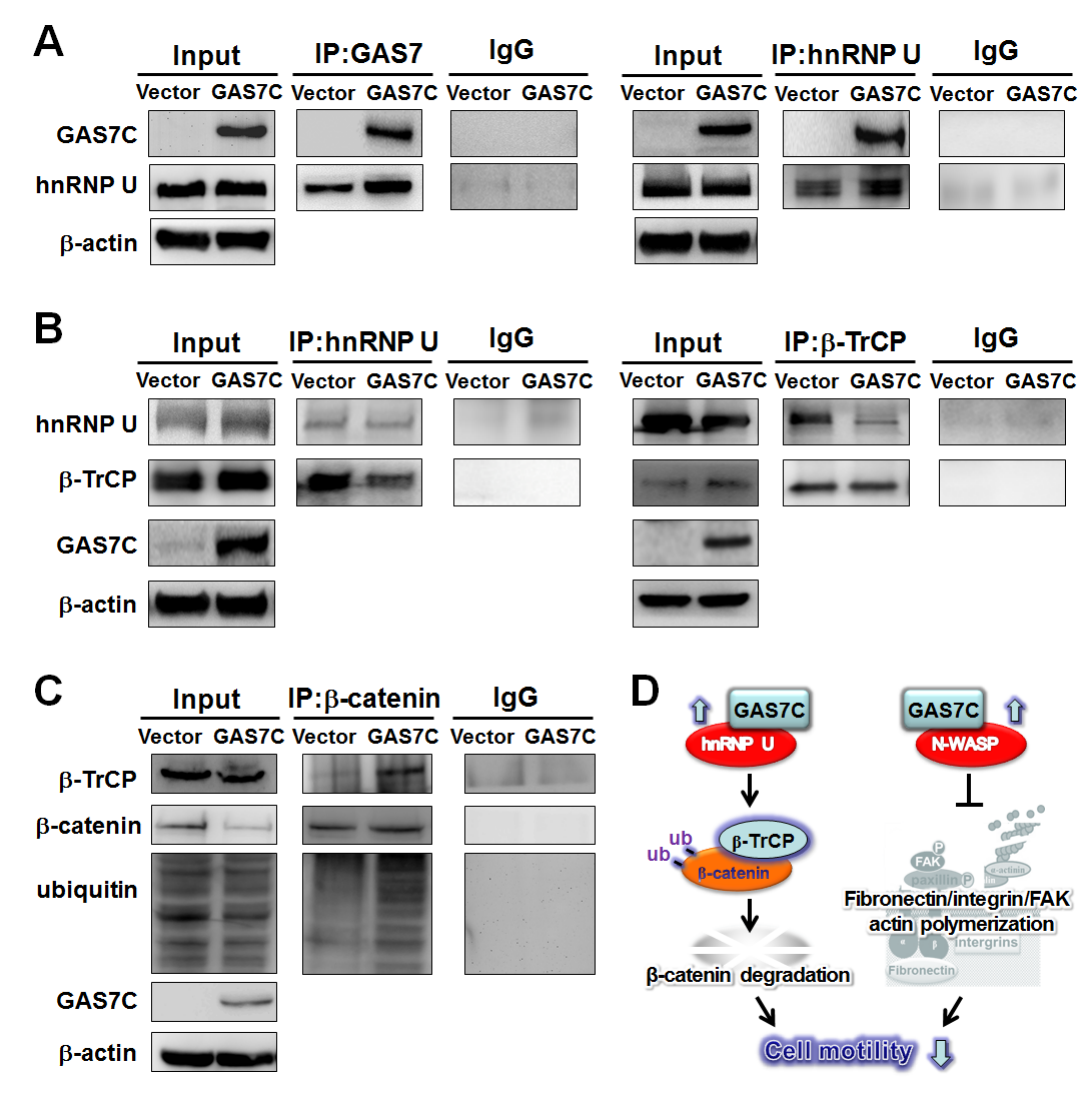
GAS7C protein is associated with hnRNP U, which is involved in the β-TrCP-mediated β-catenin degradation pathway **A.** Protein lysates of A549 cells expressing control or GAS7C vector underwent IP with anti-GAS7 (left) or anti-hnRNP U (right) antibody followed by Western blot analysis. Normal IgG served as a negative control. GAS7C and hnRNP-U were found in the same protein complex. **B.** Reduced protein-protein interactions between hnRNP U and β-TrCP in cells overexpressing GAS7C were confirmed by IP-Western blotting. **C.** The interaction between β-catenin and β-TrCP was increased along with an enhancement of ubiquitinated β-catenin level upon GAS7C overexpression. **D.** Schematic presentation using a lung model of how GAS7C inhibits cell mobility by interacting with hnRNP U to promote β-catenin degradation by β-TrCP (left) and by interacting with N-WASP to suppress fibronectin/integrin/FAK/actin polymerization (right).

hnRNP U is a shuttle protein that is able to shuttle β-TrCP between the nucleus and cytoplasm, thereby enabling β-TrCP to bind to cytoplasmic substrates such as β-catenin for ubiquitination [15, 16]. hnRNP U is another GAS7 WW domain associated protein candidate that was previously identified by proteomic screening. However, the molecular mechanism between GAS7C and hnRNP U in β-TrCP-mediated substrate degradation remains largely unclear in human cancer. To illustrate the effect of GAS7C on the interaction between hnRNP U and β-TrCP, protein lysates of A549 cells that were ectopically expressing GAS7C or vector control were subjected to IP using antibodies against GAS7, hnRNP U and β-TrCP, which was followed by Western blot analysis. The IP-Western blotting and reverse IP-Western blotting results showed that GAS7C interacted with hnRNP U (Fig. 5A), which then led to a decreased interaction between hnRNP U and β-TrCP (Fig. 5B). Next, we tested whether GAS7C overexpression increased β-TrCP-mediated β-catenin degradation. Notably, an increased interaction between β-TrCP and β-catenin was observed in cells ectopically expressing GAS7C and this was found to promote β-catenin ubiqutin-mediated degradation (Fig. 5C).

In an attempt to verify the relationship between either GAS7C/N-WASP or GAS7C/hnRNP U and cell motility in lung cancer cells, we performed siRNA knockdown of N-WASP and/or hnRNP U in A549 cells overexpressing GAS7C followed by Western blot and transwell migration assays. Our data showed that double knockdown of N-WASP and hnRNP U in A549 cells with ectopically expressed GAS7C could abolish the anti-migration effect of GAS7C overexpression (Supplementary Fig. 3C), although single knockdown of N-WASP or hnRNP U only showed partial attenuation effect (Supplementary Fig. 3A, 3B). Fig. 5D illustrates a model outlining the anti-motility effects of GAS7C by interacting with hnRNP U to sequester it from promoting β-catenin degradation by β-TrCP. In addition, GAS7C bound tightly with N-WASP to inhibit the fibronectin/integrin/FAK pathway and actin dynamics and thus to suppress cell motility. Formation of GAS7C/N-WASP or GAS7C/hnRNP U inhibited tumor cell migration via different pathways. Together, these results suggest that GAS7C cannot play a suppressive role without the presence of both N-WASP and hnRNP U.

### Analysis of the genetic and epigenetic alterations affecting the *GAS7C* gene in lung cancer patients

**Figure 6:**
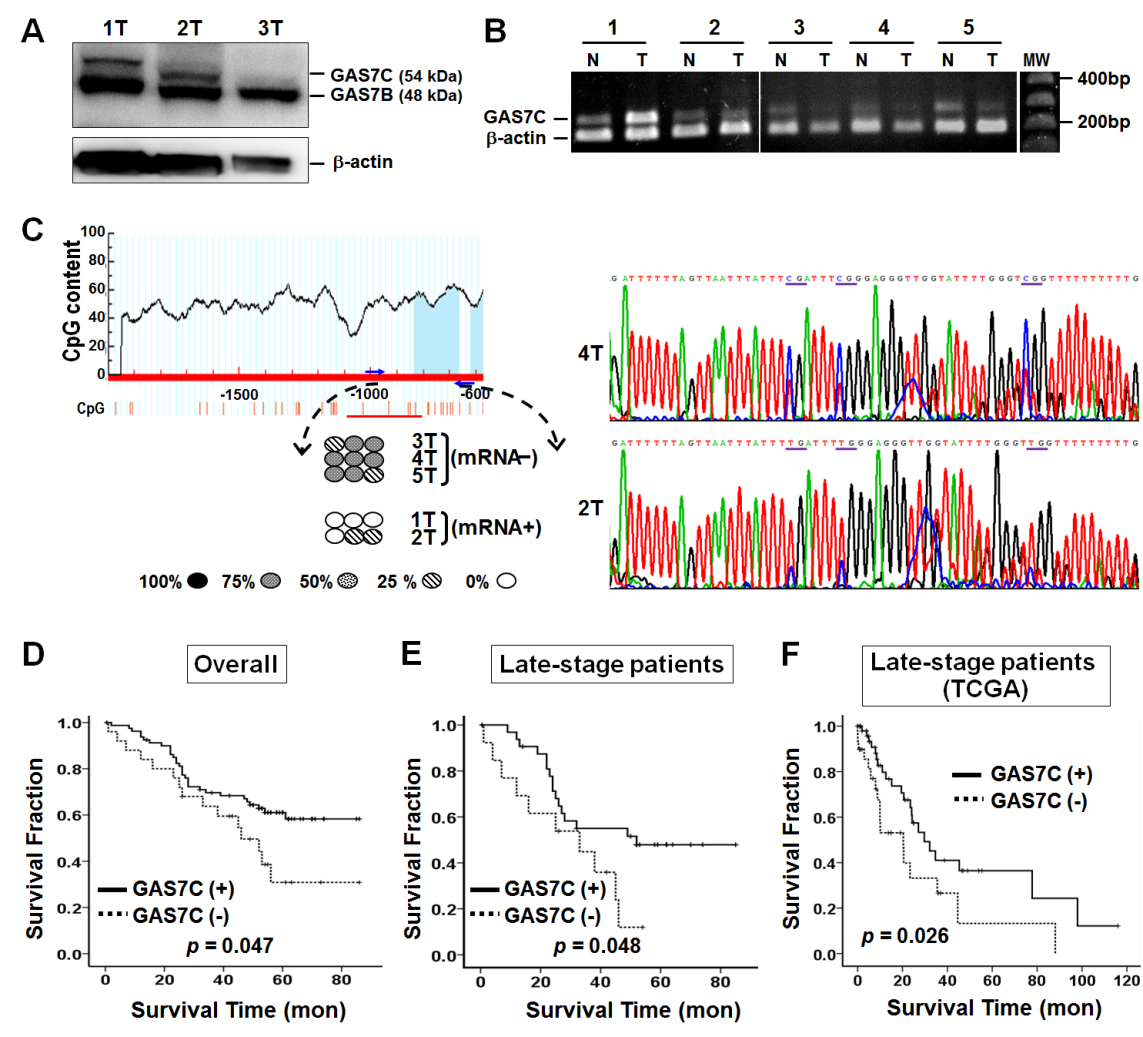
Epigenetic and survival analyses of GAS7C in lung cancer patients **A.** GAS7C and GAS7B were detected using Western blot analysis of tumor lung tissue samples. Patients 1 and 2 were positive for both GAS7C and GAS7B protein expression, whereas Patient 3 was negative for GAS7C protein expression. β-actin was used as internal control. **B.** *GAS7C* mRNA expression in the tumor tissue of Patients 1 and 2 was comparable to that of the corresponding normal tissue, whereas Patients 3, 4 and 5 had less *GAS7C* mRNA expression in their tumor tissue compared to normal tissue using the semi-quantitative RT-PCR analysis. N, normal lung tissue; T, tumor lung tissue. **C.** Methylation status of the CpG sites within the promoter region of *GAS7C* after bisulfite sequencing analysis of genomic DNA from lung cancer patients. The horizontal line indicates the genomic DNA region. Numbers below the horizontal line are the positions (in bp) within the genomic DNA. Bars under the sequences indicate the CpG sites. Methylated CpG sites at different levels are indicated as 100% 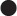, 75% 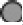, 50% 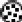, 25% 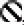, and 0% 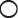. An inverse correlation between DNA methylation and mRNA expression was observed for the patients analyzed (bottom). Sequencing histograms of patient 4 with methylated CpG sequences underlined and patient 2 without methylation at the corresponding CpG sequences are shown (right). **D.**-**E.**, Kaplan-Meier survival curves with respect to reduced *GAS7C* mRNA expression for the in-house cohort. The graphs show that patients with lower *GAS7C* mRNA expression have a poorer overall survival both for all patients and for patients with late-stage cancer (stages III and IV). **F.** Low *GAS7* mRNA expression correlates with poor survival of late-stage lung cancer patients using the TCGA dataset.

Our cellular study showed that GAS7C is frequently lost in lung cancer cell lines. This result prompted us to examine whether there is lower expression of GAS7C in lung cancer patients. If this is true, what might be the underlying mechanisms of such reduced GAS7C expression? A total of 42 patients had tissue available for Western blot analysis of their GAS7C protein expression levels (Fig. 6A). The results indicated that 33.3% (14/42) of these tumors showed absent or low expression of GAS7C protein. Next, a semi-quantitative RT–PCR analysis was conducted to explore the mRNA expression level of the *GAS7C* gene in 106 lung cancer patients (Fig. 6B). *GAS7C* transcripts were found to be decreased or undetectable in 23.6% (25/106) of these lung cancer patients. Notably, low levels of *GAS7C* transcription were found in 60.0% of patients with metastatic cancer (p = 0.054, Table 1).

**Table 1:** Correlation between GAS7C mRNA expression, clinicopathological parameters and DNA methylation status of lung cancer patients

		*GAS7C* mRNA			
Characteristics		Total ^a^	− (%)	+	*p* value ^b^
Overall		106	25 (23.6)	81	
Gender	male	76	18 (23.7)	58	0.969
	female	30	7 (23.3)	23	
Smoking habit	smoker	30	7 (23.3)	23	0.864
	nonsmoker	76	18 (23.7)	58	
Tumor type ^c^	ADC	66	17 (25.8)	49	0.408
	SCC	28	5 (17.9)	23	
Tumor stage	I	44	8 (18.2)	36	0.086
	II	16	4 (25.0)	12	
	III	42	10 (23.8)	32	
	IV	4	3 (75.0)	1	
Metastasis	M0	99	22 (22.2)	77	**0.054**
	M1	5	3 (60.0)	2	
*GAS7C* promoter hypermethylation	−	23	3 (37.5)	20	**0.013**
	+	8	5 (62.5)	3	

a The total number of sample in some categories is less than the overall number analyzed because clinical data or promoter methylation data was not available for these samples.

b Bold values indicate statistical significance.

c ADC: adenocarcinoma; SCC: squamous cell carcinoma.

Given that promoter hypermethylation is able to produce transcriptional silencing of a target gene [23], we further examined the promoter methylation of the *GAS7C* gene in lung cancer patients. To do this, we first searched for the promoter region of *GAS7C* by characterizing the regulatory sequence using a luciferase reporter assay. We found that *GAS7C* promoter activity was mainly derived from the region −1245 bp to −496 bp upstream from the transcription start site (Supplementary Fig. 4). Next, we performed bisulfite sequencing of this *GAS7C* promoter region in 36 available tumor tissue samples (Fig. 6C). The results showed that promoter hypermethylation of the *GAS7C* gene occurred in 16.7% (6/36) of the tumors analyzed. Of note, methylation of several hot spot CpGs correlated with the mRNA expression status of the samples (circle symbols in Fig. 6C). In addition, low mRNA expression was found to be significantly associated with promoter hypermethylation (*p* = 0.013, Table 1). To determine whether *GAS7C* promoter methylation is the predominant mechanism causing loss of *GAS7C* gene expression, A549 cells were treated with the DNA demethylation agent 5-aza-dC and then subjected to RT-PCR and Western blot analysis. A549 cells treated with 5-aza-dC were found to have successfully restored levels of GAS7C mRNA and protein expression (Supplementary Fig. 5).

### Correlation between the expression of *GAS7C* and the prognosis of lung cancer patients

Our cell and animal studies have shown that overexpression of GAS7C is able to attenuate lung cancer cell motility and tumor metastasis. Therefore, we hypothesized that *GAS7C* low expression may predict a poorer survival among lung cancer patients. To evaluate the prognostic effects of low *GAS7C* mRNA expression, survival curves were created using the Kaplan-Meier method for our cohort of 106 lung cancer patients. Interestingly, the results showed that low or undetectable *GAS7C* mRNA expression was clearly associated with a poorer survival for all patients (*p* = 0.047) and for patients with late-stage cancer (stages III and IV) (*p* = 0.048) (Fig. 6D-6E).

We further performed a prognosis analyses in relation to *GAS7* mRNA expression using publicly available microarray data of non-small cell lung cancer samples from TCGA (The Cancer Genome Atlas). This database includes gene expression data obtained from 82 late-stage lung cancer samples (Supplementary Table 1). As shown in Fig. 6F, a low level of *GAS7* mRNA expression is associated with a poorer survival among these patients with late-stage lung cancer from the TCGA database (*p* = 0.026). These findings further support the hypothesis that a low level of GAS7 expression could be a useful prognostic factor for lung cancer.

## DISCUSSION

GAS7 is a member of the growth-arrest-specific family that is preferentially expressed during cell growth arrest or G0 stage [24] and it was originally isolated from serum-starved mouse NIH 3T3 cells [4]. The human *GAS7* gene has been shown to encode three isoforms (GAS7A, GAS7B, and GAS7C) that are normally expressed in the brain and are involved in neuritogenesis in mammals [6]. However, the expression and function of GAS7 isoforms during tumorigenesis remains unknown.

In the present study, we report for the first time that GAS7C is expressed in normal lung tissue but is frequently down-regulated in lung cancer cells and lung tumor tissues. In an effort to better understand the mechanism associated with low GAS7C expression in lung cancer patients, we carried out a comprehensive molecular analysis including mRNA/protein expression, DNA methylation and allelic imbalance of the *GAS7C* gene. Allelic loss of either AFMA070WD1 or D17S945 in the *GAS7* locus was found in 17.1% of 70 informative cases (Supplementary Fig. 6). However, low mRNA expression was not significantly associated with allelic imbalance partly due to the fact that allelic loss contributes to only one “hit” among the multiple-hit model for cancer development [25]. Our results also revealed that there was a correlation between decreased mRNA expression and promoter hypermethylation of *GAS7C* gene. We also observed that a low level of expression of *GAS7C* mRNA was significantly associated with a poorer survival in both Asian and Caucasian lung cancer patients. Mechanistically, ectopically expression of GAS7C was found to suppress lung cancer cell migration by inactivating the fibronectin/integrin/FAK/F-actin/N-WASP pathway, which is involved in cytoskeleton rearrangements. In addition, GAS7C is able to sequester hnRNP U, which enables β-TrCP/β-catenin complex formation leading to β-catenin degradation (Fig. 5D). The reconstitution experiments and cell migration assays confirm the causal relationship of GAS7C in sequestering N-WASP and hnRNP U to achieve the anti-migration effect (Supplementary Fig. 3). Our results suggest that *GAS7C* is a candidate tumor suppressor gene in lung tumorigenesis. These findings provide new evidence supporting the hypothesis that down-regulation of GAS7C leads to lung cancer progression.

Previous studies have demonstrated that overexpression of GAS7 increases nerve growth factor-mediated neuronal differentiation of pheochromocytoma PC12 cells [26]. It has also been reported that GAS7 colocalizes with F-actin [27] and that GAS7B directly interacts with N-WASP to regulate neurite outgrowth [10]. In addition, interactions between of GAS7B and G-actin/tubulin promote microtubule bundling to alter the cytoskeletal organization of neuron cells [28]. These studies indicate that GAS7B not only plays a role in neuron differentiation but that it is also able to associate with microfilaments, allowing regulation of the cytoskeleton dynamics. Therefore, we further examined the association of the N-WASP protein with GAS7B in lung cancer cells that were ectopically expressing GAS7B. Our results showed that overexpression of GAS7B increased the association between GAS7B and N-WASP as evidenced by the IP-Western blot assays (Supplementary Fig. 7). N-WASP is a major regulator of actin polymerization. We will further confirm whether down-regulation of the fibronectin/integrin/FAK pathway is also involved in GAS7B/N-WASP actin dynamic to decrease lung cancer cell motility.

Furthermore, our IP-Western blotting results suggest that GAS7C interacts with hnRNP U, a pseudo-substrate of E3-ubiqutin ligase β-TrCP. The GAS7C/hnRNP U interaction results in the release of β-TrCP and this will facilitate the β-TrCP/β-catenin interaction, which increases the ubiquitination of β-catenin; this in turn will result in a reduction in the level of β-catenin protein (Fig. 5). It is known that β-catenin at the adherent junctions plays an anchoring role in the actin cytoskeleton [18, 29]. It has been long recognized that a loss of fibronectin from the extracellular matrix is a hallmark of tumor malignancy [30]. Fibronectin degradation is ubiquitination-dependent and is controlled by β-TrCP [31]. Importantly, our findings show that GAS7C overexpression dramatically reduces the amount of fibronectin present on the cell membrane (Fig. 4C), which suggests the possibility of promotion of fibronectin degradation by GAS7C. Collectively, these findings suggest that GAS7C overexpression decreases the migration of lung cancer cells at least partly through the fibronectin/integrin/FAK/N-WASP actin dynamics pathway and the hnRNP U/β-TrCP/β-catenin ubiquitin-degradation pathway.

The growth-arrest-specific genes family includes *GAS1, GAS2, GAS3, GAS5, GAS6, GAS7, GAS8*, and *GAS9*. They are known to be involved in cell cycle control, suggesting a potential role in regulating cell proliferation [32]. However, we observed that GAS7C overexpression did not change the cell cycle distribution either in the presence of absence of a cell cycle arrest inducer, namely ultraviolet light treatment (Supplementary Fig. 8). These results suggested that GAS7C may not play a major role in cell cycle regulation in lung cells. Similarly, overexpression of another member of the GAS family, *GAS5*, did not change the cell cycle distribution of the MCF-10A human breast cancer cell line and a mouse thymoma cell line [33]. *GAS5* is a non-protein coding gene but does encoded several small-nucleolar-RNA (snoRNA) in its introns [34]. Notably, Gas7 has been shown to play a role in cellular migration in the embryonic stem cells of mice [35]. These results suggested that the members of the growth-arrest-specific family may exert various functions in cell physiology.

In conclusion, we provide novel findings on the anti-cell motility function of GAS7C and how alteration in GAS7C expression affects lung cancer. Using cell-based and animal studies, we have demonstrated that overexpression of GAS7C not only decreases migration and invasion abilities of lung cancer cells *in vitro* but also attenuates the potential of lung cancer metastasis *in vivo*. In clinical studies, we have identified a correlation between a low level of *GAS7C* mRNA expression, promoter hypermethylation and the prognosis of lung cancer patients. Low *GAS7C* mRNA expression is frequently detected in lung cancer samples, for example in 75.0% of stage IV patients and in 60.0% of metastatic patients (Table 1), indicating an association between a low level of *GAS7C* mRNA expression and cancer progression. Consistently, a low level of *GAS7C* expression correlates with a poorer survival stratified using late-stage lung cancer from Asian and Caucasian populations (Fig. 6E-6F), which supports the idea that *GAS7C* may serve as a prognostic biomarker in lung cancer patients with metastasis. Furthermore, it is possible that GAS7C may also act as a metastasis suppressor in other cancers. Therapeutic strategies, such as DNA demethylation of the *GAS7C* gene as well as approaches that increase the stability of the GAS7C protein may help with the development of therapies that are able to target lung cancer.

## MATERIALS AND METHODS

### Cell culture

The normal human lung cell lines, namely Beas2B and MRC5, as well as the human lung cancer cell lines, namely A549, H226, H226Br, H460 and H1299, were purchased from the American Tissue Culture Company. The isogenic human lung cancer cell lines CL1-0 and CL1-5 with low and high motility, respectively, were provided by Dr. Pan-Chyr Yang at Department of Internal Medicine, National Taiwan University, Taiwan [36]. All cell lines were maintained in Dulbecco's Modified Eagle Medium (DMEM, pH7.4, Gibco, Invitrogen, Carlsbad, CA) with 10% Fetal Bovine Serum (FBS, Gibco) and 1% penicillin/streptomycin (100 units/ml penicillin and 100 μg/ml streptomycin, Gibco). All cell lines were incubated at 37oC in a humidified atmosphere of 5% CO_2_/95% air.

### Ectopically expressed GAS7C analysis

The pCMV6-XL5 and pCMV6-XL-GAS7C plasmids were purchased from OriGene Company (OriGene technologies, MD). The transfections were carried out using ExGen 500 (MBI Fermentas, Flamborough, Ontario, Canada) at a ratio of 3.3 μl of ExGen 500 per 1 μg of DNA according to the manufacturer's protocol.

### Knockdown analysis

The siRNA sequences of GAS7, N-WASP, and hnRNP U are as described in Supplementary Table 2. Cells (1×10^5^) were transfected with 5 μg of siRNA using ExGen 500 transfection reagent (Fermentas) as recommended by the manufacturer.

### Western blotting

Samples containing equal amounts of protein (50 μg) were separated on an 8% SDS-PAGE and then electroblotted onto Immobilon-P membranes (Millipore Co., Bedford, MA). Immunoblot analysis was performed using the appropriate primary antibody [GAS7, FAK, p-FAK, paxillin, p-Paxillin, integrin β1, Cytokeratin 7, fibronectin, F-actin (Alexa-Fluor 568 phalloidin), DAPI, N-WASP, hnRNP U, β-catenin, β-TrCP, IgG, p-p53, or β-actin] at 4oC overnight. Antibodies and the conditions used are described in Supplementary Table 3. Each Western blot analysis was repeated three times.

### Cdc42 activation assay

The Cdc42 activation assay was performed using the Cdc42 Activation Assay Kit (Millipore, Bedford, MA) according to the manufacture's instruction.

### Immunocytochemistry staining

Suspended cells (1 × 10^4^) seeded onto chamber slides were hybridized with anti-fibronectin (1:600, Sigma, Louis, MO). Pericellular poly-fibronectin assemblies were photographed using bright field and fluorescent microscopy (Olympus FV1000 confocal microscope).

### Immunofluorescent staining

Cells were fixed with 1% paraformaldehyde and permeabilized with 0.1% Triton X-100. The cells were then incubated with primary antibody at 4°C overnight, followed by washing with PBS and incubation with phalloidin-conjugated (red fluorescence, representing F-actin) or FITC-conjugated (green fluorescence, representing GAS7) secondary antibody together with DAPI (to stain the nucleus) at 4°C for 1 hr. Immunofluorescence was visualized using confocal microscopy (FV1000; Olympus).

### Immunoprecipitation (IP)

For immunoprecipitation, 2000 μg cell protein lysates were incubated with 5 μg of the appropriate antibody as described in Supplementary Table 3. Next, 10 μl of affinity ligand was added followed by 1× wash buffer to make a final volume of 500 μl using the kit from Catch and Release Reversible Immunoprecipitation System (Upstate Biotechnology, Inc., Lake Placid, NY). After incubation at 4oC overnight, the immune complexes were washed three times with 1× immunoprecipitation buffer. Proteins were eluted by boiling in 2× sample buffer, followed by separation by 8% SDS-PAGE and Western blot analysis using the appropriate antibodies.

### Transwell migration and invasion assays

Transwell assays were performed to determine the migration and invasion ability of GAS7C overexpression cells and vector control cells. A similar analysis was also performed on GAS7C knockdown cells and si-control cells. The transwell (Falcon, BD Labware, Bedford, MA) consists of upper and lower chambers separated by a layer of Millipore membrane with pore size of 8μm. About 5 × 10^5^ cells were seeded into the upper chamber with or without Matrigel (2.5 mg/ml, Sigma) and cultured for 20 hours. Cells attached on the reverse side of the membrane were then fixed and stained. Six randomly selected fields were photographed and quantified. The experiment was carried out three times.

### Wound healing assay

The wound healing assay were carried out using a Culture-insert (Ibidi) on A549, H226Br, CL1-0, and H1299 cells transfected with either an expression plasmid or an appropriate si-RNA oligo for GAS7C. A cell-free gap of 500 μm was created after removing the Culture-insert. The remaining cell-free gap area was determined using ImageJ software after culturing for 8-16 hours. The widths of the remaining open wound were measured. Three independent experiments were performed.

### Experimental metastasis animal model

Female BALB/c nude mice, 5-6 weeks of age, were obtained from the National Laboratory Animal Center (Taipei, Taiwan) and raised in a pathogen-free environment. About 1 × 10^6^ A549 lung cancer cells in 200 μl PBS were injected via the tail vein for the metastasis analysis. Mice were sacrificed at 5 weeks after injection and the lung tissues from the mice were resected, fixed and embedded in paraffin for histological H&E staining and analysis by pathologists (Department of Pathology, National Cheng-Kung University Hospital, Tainan, Taiwan).

### Patient information and clinical samples

Paired tumor and normal lung tissues were obtained from 106 lung cancer patients recruited at the Taipei Veterans General Hospital between 2002 and 2008 after obtaining appropriate institutional review board permission and informed consent. Overall survival was calculated from the day of surgery to the date of death or the last follow-up. For the RNA expression assay, total RNA was extracted from 106 paired tumor and normal tissues using Trizol reagent (Invitrogen). cDNA was synthesized using SuperScript^TM^ reverse transcriptase (Invitrogen) according to the manufacturer's instructions. For the methylation assay, genomic DNA from 31 available tumor tissue samples was extracted using proteinase K digestion and phenol–chloroform extraction. High quality protein for Western blot analysis was extracted from 42 available tumor samples.

### Semi-quantitative reverse-transcriptase polymerase chain reaction (RT-PCR) analysis

*GAS7C* mRNA expression were measured by multiplex RT–PCR analysis using the *β-actin* gene as an internal control. The primers for the RT-PCR were as follows: GAS7C-F 5′- CGAGCTACGTGCAGTTGCT -3′; GAS7C-R 5′ -CATGTGGGCAGTCTCTGGAG- 3′; β-actin-F 5′- GGCGGCACCACCATGTACCCT -3′; β-actin-R 5′- AGGGGCCGGACTCGTCATACT -3′. Reactions were carried out in a final volume of 25 μl with 1 μl of cDNA and 0.25 *p*mol of primers in a DNA Thermal Cycler (PE Applied Biosystems, Foster City, CA). The relative levels of gene expression were calculated as previously described [37].

### Bisulfite sequencing analysis

Bisulfite sequencing PCR analyses were first conducted using primers targeting the *GAS7C* promoter region, sequences of the primers were as follows: outer sense primer 5′- TTTATGGAGTAGATTAGTAAAGAAATTAAG -3′ and outer antisense primer 5′- CAAAAAAACCTATCACTAAAAAAAA 3′; inner sense primer 5′- TGGAGAGTAGGGATTGGTTTAAATGAT -3′ and inner antisense primer 5′- CCTAAACCCAACAACCTAATAACAAC -3′. The PCR products were then sequenced using the inner forward and reverse primers on an ABI Prism 377 DNA Sequencer (PE Applied Biosystems).

### Statistical analysis

The Pearson's χ^2^ test was used to compare the changes in *GAS7C* mRNA expression across the various lung cancer patients at different disease stages. Overall survival curves were calculated according to the Kaplan-Meier method, and comparison was performed using the log-rank test. The two-tailed Student's t-test was used for the cell and animal studies. Each experiment was repeated at least three times and is represented as the mean ± SD. A value *p* < 0.05 was considered to be statistically significant.

## SUPPLEMENTARY MATERIALS TABLES AND FIGURES


