# Supplementary material for: Growth-arrest-specific 7C protein inhibits tumor metastasis via the N-WASP/FAK/F-actin and hnRNP U/β-TrCP/β-catenin pathways in lung cancer
Source: Oncotarget. 2015 Oct 25;6(42):44207–21. doi: 10.18632/oncotarget.6229 (PMC4792552; doi:10.18632/oncotarget.6229)
Supplement: Supplementary file 1 [file oncotarget-06-44207-s001.pdf]

Growth-arrest-specific 7C protein inhibits tumor metastasis via the N-WASP/FAK/F-actin and hnRNP U/β-TrCP/β-catenin pathways in lung cancer

Supplementary Materials

Materials and Methods

Isoform-specific PCR analysis

cDNA panels for different human tissues were purchased from Clontech (Palo Alto, CA). Isoform-specific PCR primers (See PCR condition in main text) were used to amplify *GAS7C* specifically. The relative levels of *GAS7C* mRNA expression in various tissues were calculated and compared across lung tissue samples.

Luciferase assay

The *GAS7C* promoter (848 bp or 750 bp) was amplified by PCR using human genomic DNA as a template. Primers of *GAS7C* for the 848 bp were: 848F 5'-AAACTTCATTGGGCCAGCTA -3'; 848R 5'- CGAATGGCTCACTTGAGGTC -3'; 750F 5'- GATGGGGTTTCACCTTGTTG -3'; 750R 5'-TCTGTTCTGTGCCTGCTTTG -3'. Reactions were carried out in a volume of 25 μl with 1 μl of cDNA and 0.25 *p*mol of primers in a DNA Thermal Cycler. The promoter was subcloned into the pGL4-luciferase vector using *Sac*I and *Xho*I restriction sites. All of the constructs were sequenced. The luciferase activities were measured using the Dual-Glo Luciferase Assay System (Promega).

5-aza-2'-deoxycytidine (5-aza-dC) treatment of lung cancer cells

The cells were treated with 50 μM 5-aza-dC (Sigma-Aldrich, MO) for three doubling times. As DNA replication progresses, the 5-aza-dC nucleoside inhibitor that is

incorporated into the DNA will reduce DNA methylation. The cells were then harvested for RT-PCR and Western blot assays.

Loss of heterozygosity (LOH) assay

Genomic DNA (20 ng) from normal lung cells or tumor samples were used for each PCR analysis. The microsatellite markers located near the *GAS7* loci were AFMA070WD1 and D17S945. PCR products were mixed with fluorescent molecular weight markers for subsequent electrophoresis in a MegaBACE 1000 automatic sequencer (Amersham Pharmacia, Piscataway, NJ). Allele sizes were determined using Genetic Profiler Analysis version 2.0 software. The allelic ratio was calculated as previously described [2].

Analysis of cell cycle distribution

Lung cancer cells A549 were transfected with GAS7C plasmid or vector control for 48 hours. Adherent and floating cells were collected, washed once with PBS, and fixed with ice-cold 90% ethanol at least overnight at –20°C until analysis. Fixed cells were collected by centrifugation and washed once with PBS to remove any ethanol. Cells were resuspended in 1 ml of PBS mixture [contains 20 μg/ml propidium iodide (PI, Sigma-Aldrich), 200 μg/ml RNase A, and 1 μl Triton X-100, preheated at 37°C for 20 minutes] and then incubated at 37°C in the dark for 15 minutes. The cell cycle distribution was determined using a FACScan Flow cytometer (BD, Franklin Lakes, NJ) and calculated using ModFIT LT 2.0 version software (BD).

Supplementary Table 1. TCGA data of *GAS7* mRNA expression and clinical follow-up.

CASE ID	Histology	Tumor Stage	Survival (Months)	Survival Status	GAS7 [a]
TCGA-05-4384	Lung Adenocarcinoma	Stage IIIA	14	LIVING	-0.1724
TCGA-05-4395	Lung Adenocarcinoma	Stage IIIB	0	DECEASED	-1.0583
TCGA-05-4396	Lung Adenocarcinoma	Stage IIIB	9.96	DECEASED	-1.0366
TCGA-05-4398	Lung Adenocarcinoma	Stage IIIB	47	LIVING	-0.4738
TCGA-05-4402	Lung Adenocarcinoma	Stage IV	8.02	DECEASED	0.0337
TCGA-05-4415	Lung Adenocarcinoma	Stage IIIB	2.96	DECEASED	-1.2091
TCGA-05-4418	Lung Adenocarcinoma	Stage IIIA	8.97	DECEASED	-0.5274
TCGA-05-5420	Lung Adenocarcinoma	Stage IIIA	1.02	LIVING	-0.4819
TCGA-05-5429	Lung Adenocarcinoma	Stage IIIA	0.986	LIVING	-1.1456
TCGA-38-4632	Lung Adenocarcinoma	Stage IV	44.6	DECEASED	-0.9576
TCGA-38-6178	Lung Adenocarcinoma	Stage IIIA	5.19	LIVING	0.8041
TCGA-44-3396	Lung Adenocarcinoma	Stage IIIA	10.2	LIVING	1.1332
TCGA-44-6774	Lung Adenocarcinoma	Stage IIIA	5.42	LIVING	3.035
TCGA-49-4490	Lung Adenocarcinoma	Stage IIIA	12.6	DECEASED	-0.2119
TCGA-49-4494	Lung Adenocarcinoma	Stage IIIA	35.5	DECEASED	-1.2435
TCGA-49-4507	Lung Adenocarcinoma	Stage IIIA	8.81	DECEASED	-0.9857
TCGA-49-4512	Lung Adenocarcinoma	Stage IIIA	29.7	DECEASED	0.4535
TCGA-49-6745	Lung Adenocarcinoma	Stage IIIA	4.93	LIVING	-0.0357
TCGA-49-6761	Lung Adenocarcinoma	Stage IIIA	0.0329	LIVING	-0.3362
TCGA-50-5044	Lung Adenocarcinoma	Stage IIIB	20.5	DECEASED	-0.7058
TCGA-50-5051	Lung Adenocarcinoma	Stage IIIA	14.9	LIVING	-0.9727

TCGA-50-5072	Lung Adenocarcinoma	Stage IIIA	8.05	LIVING	0.2578
TCGA-50-5933	Lung Adenocarcinoma	Stage IIIB	77.8	DECEASED	1.1529
TCGA-50-5936	Lung Adenocarcinoma	Stage IIIA	8.44	DECEASED	0.259
TCGA-50-5941	Lung Adenocarcinoma	Stage IIIA	9.36	LIVING	0.7424
TCGA-50-6593	Lung Adenocarcinoma	Stage IIIA	11	DECEASED	0.6318
TCGA-50-6595	Lung Adenocarcinoma	Stage IIIA	6.21	DECEASED	-0.6423
TCGA-53-7813	Lung Adenocarcinoma	Stage IIIB	13.7	LIVING	-1.0679
TCGA-55-1594	Lung Adenocarcinoma	Stage IIIA	38.7	LIVING	-0.5761
TCGA-55-6970	Lung Adenocarcinoma	Stage IIIA	15.2	DECEASED	-0.1315
TCGA-55-6981	Lung Adenocarcinoma	Stage IIIA	45.3	DECEASED	-0.512
TCGA-55-7727	Lung Adenocarcinoma	Stage IIIA	1.02	LIVING	0.2144
TCGA-64-1677	Lung Adenocarcinoma	Stage IIIA	20.6	DECEASED	0.6582
TCGA-64-1679	Lung Adenocarcinoma	Stage IIIA	55.4	LIVING	-0.1036
TCGA-64-1680	Lung Adenocarcinoma	Stage IV	37	LIVING	-1.0694
TCGA-64-5775	Lung Adenocarcinoma	Stage IIIA	0.0986	DECEASED	-0.7025
TCGA-69-7765	Lung Adenocarcinoma	Stage IV	4.24	LIVING	0.6052
TCGA-73-4659	Lung Adenocarcinoma	Stage IIIA	23.4	DECEASED	-0.961
TCGA-73-4675	Lung Adenocarcinoma	Stage IIIA	1.31	LIVING	0.1157
TCGA-78-7145	Lung Adenocarcinoma	Stage IV	27.1	DECEASED	-0.5276
TCGA-78-7149	Lung Adenocarcinoma	Stage IIIB	35.9	LIVING	-1.3066
TCGA-78-7154	Lung Adenocarcinoma	Stage IIIA	19.5	DECEASED	0.3799
TCGA-78-7156	Lung Adenocarcinoma	Stage IV	32.1	DECEASED	-0.0578
TCGA-78-7158	Lung Adenocarcinoma	Stage IIIB	5.88	DECEASED	-1.3892

TCGA-78-7160	Lung Adenocarcinoma	Stage IV	22.3	LIVING	-0.651
TCGA-78-7167	Lung Adenocarcinoma	Stage IV	88.1	DECEASED	-0.7901
TCGA-78-7536	Lung Adenocarcinoma	Stage IIIA	8.02	DECEASED	-1.2365
TCGA-86-7714	Lung Adenocarcinoma	Stage IIIA	0	LIVING	-1.3656
TCGA-91-6849	Lung Adenocarcinoma	Stage IIIA	0.92	LIVING	-0.8878
TCGA-97-7554	Lung Adenocarcinoma	Stage IIIA	8.64	LIVING	-0.0238
TCGA-99-7458	Lung Adenocarcinoma	Stage IIIA	8.21	LIVING	-1.1062
TCGA-18-3410	Lung Squamous Cell Carcinoma	Stage IIIA	4.8	DECEASED	-0.819
TCGA-18-3411	Lung Squamous Cell Carcinoma	Stage IIIA	46.5	LIVING	-0.6703
TCGA-18-3414	Lung Squamous Cell Carcinoma	Stage IV	23.5	DECEASED	-0.1004
TCGA-18-3417	Lung Squamous Cell Carcinoma	Stage IV	24.9	LIVING	0.5723
TCGA-21-1070	Lung Squamous Cell Carcinoma	Stage IIIA	116	LIVING	0.4224
TCGA-22-4591	Lung Squamous Cell Carcinoma	Stage IIIA	20.4	DECEASED	-1.0896
TCGA-22-4595	Lung Squamous Cell Carcinoma	Stage IIIA	24.1	DECEASED	1.0874
TCGA-22-4601	Lung Squamous Cell Carcinoma	Stage IIIA	34.7	DECEASED	-0.1518
TCGA-33-4538	Lung Squamous Cell Carcinoma	Stage IIIA	98	DECEASED	-0.4234
TCGA-37-3783	Lung Squamous Cell Carcinoma	Stage IIIA	3.94	LIVING	-0.1197
TCGA-37-4133	Lung Squamous Cell Carcinoma	Stage IIIB	7.82	LIVING	-0.4816
TCGA-37-4135	Lung Squamous Cell Carcinoma	Stage IIIB	6.8	LIVING	-0.7121
TCGA-39-5029	Lung Squamous Cell Carcinoma	Stage IIIA	24.3	DECEASED	0.2871
TCGA-39-5030	Lung Squamous Cell Carcinoma	Stage IIIA	1.94	DECEASED	1.6378
TCGA-46-3768	Lung Squamous Cell Carcinoma	Stage IIIA	9.95	DECEASED	-0.7018
TCGA-51-4080	Lung Squamous Cell Carcinoma	Stage IIIB	0.39	DECEASED	-0.8865

TCGA-60-2724	Lung Squamous Cell Carcinoma	Stage IIIA	1.22	LIVING	0.2221
TCGA-66-2742	Lung Squamous Cell Carcinoma	Stage IV	21.1	LIVING	-0.5693
TCGA-66-2754	Lung Squamous Cell Carcinoma	Stage IIIA	2	LIVING	-1.8284
TCGA-66-2756	Lung Squamous Cell Carcinoma	Stage IIIB	0.99	LIVING	-1.427
TCGA-66-2759	Lung Squamous Cell Carcinoma	Stage IIIA	25	LIVING	0.1342
TCGA-66-2766	Lung Squamous Cell Carcinoma	Stage IIIA	1.02	LIVING	-0.7457
TCGA-66-2767	Lung Squamous Cell Carcinoma	Stage IIIB	2	LIVING	0.1493
TCGA-66-2778	Lung Squamous Cell Carcinoma	Stage IIIB	19	LIVING	-1.4484
TCGA-66-2783	Lung Squamous Cell Carcinoma	Stage IIIB	24.9	LIVING	-0.0921
TCGA-66-2789	Lung Squamous Cell Carcinoma	Stage IIIB	4.04	DECEASED	-0.2549
TCGA-66-2791	Lung Squamous Cell Carcinoma	Stage IIIB	5.03	DECEASED	0.7102
TCGA-66-2793	Lung Squamous Cell Carcinoma	Stage IIIB	10.1	DECEASED	-0.9231
TCGA-66-2794	Lung Squamous Cell Carcinoma	Stage IIIB	54	LIVING	-0.1923
TCGA-66-2795	Lung Squamous Cell Carcinoma	Stage IIIB	4.01	LIVING	-0.1133
TCGA-66-2800	Lung Squamous Cell Carcinoma	Stage IIIB	49	LIVING	-0.162

[a] GAS7 Z-score $\leq$ - 0.8 (1SD) indicates "low expression".

Supplementary Table 2. siRNA sequences used in the present study.

RNAi	Function	Double-stranded RNA sequences (5' → 3')	Source
GAS7C	Knockdown GAS7C	5'-GCUCGGCGGGAAUGCAGGCUGCGCU-3'<br>5'-AGCGCAGCCUGCAUUCCCGCCGAGC-3'<br>5'-UUCACAGCGCAGCCUGCAUUCCCGC-3'	Invitrogen
N-WASP	Knockdown N-WASP	5'-TGGAATCTTAGAGGCACAACTTAAA-3'<br>5'-AAACAGGAGGTGTTGAAGCTGTTAA-3'<br>5'-AGATACGACAGGGTATCCAACTAAA-3'	TAQKEY Science
hnRNP U	Knockdown hnRNP U	5'-CAAATCTCCTCAGCCACCTGTTGAA-3'<br>5'-CACAGTGGTTTGTCTTGATACTTAT-3'<br>5'-GAGAAGATCCCAGTAAGGCATTTAT-3'	TAQKEY Science

Supplementary Table 3. The antibodies and their reaction conditions used in the present study.

Target	kDa	Raised In	Application	Dilution	Source	Catalog No.
GAS7 [a]	38, 48, 54	Rabbit	Western blot	1:500	Proteintech	10072-1-AP
GAS7 [a]	38, 48, 54	Goat	Western blot	1:500	Santa Cruz	sc-34364
			Immunofluorescence	1:500		
FAK	125	Mouse	Western blot	1:500	Abcam	ab55632
FAK phosphor Y397	125	Mouse	Western blot	1:250	Abcam	ab24781
paxillin	64	Mouse	Western blot	1:500	Abcam	ab3127
paxillin phosphor Y31	64	Rabbit	Western blot	1:500	Abcam	ab32115
integrin β1	130	Mouse	Western blot	1:5000	Novus	610468
Cytokeratin 7	51	Rabbit	Western blot	1:1000	GeneTex	GTX110414
Fibronectin	220	Rabbit	Fibronectin assembly assay	1:600	Sigma	F3648
F-actin (Alexa-Fluor 568 phalloidin)	-- [b]	Amanita phalloides mushroom	Immunofluorescence	1:200	Invitrogen	A12380
DAPI	-- [b]	-- [c]	Immunofluorescence	1:5000	Sigma	D8417
N-WASP	65	Rabbit	Western blot	1:500	Santa Cruz	sc-20770
			Immunoprecipitation			
hnRNP U	143	mouse	Western blot	1:500	Santa Cruz	sc-32315
			Immunoprecipitation			
β-catenin	92	mouse	Western blot	1:700	Abcam	ad6302
			Immunoprecipitation			
β-TrCP	70	mouse	Western blot	1:500	invitrogen	37-3400
			Immunoprecipitation			
IgG	150	Mouse	Immunoprecipitation	1:1000	Millipore	12-371

β-actin	42	Mouse	Western blot	1:5000	Novus	NB 600-501
p53 phosphor S15	54	Rabbit	Western blot	1:500	Abcam	ab38497

[a] The molecular weight of GAS7 isoforms are 38 kDa, 48 kDa, and 54 kDa for GAS7A, GAS7B, and GAS7C, respectively.

[b] --: Molecular weight is variable.

[c] --: It is used for nuclear staining.

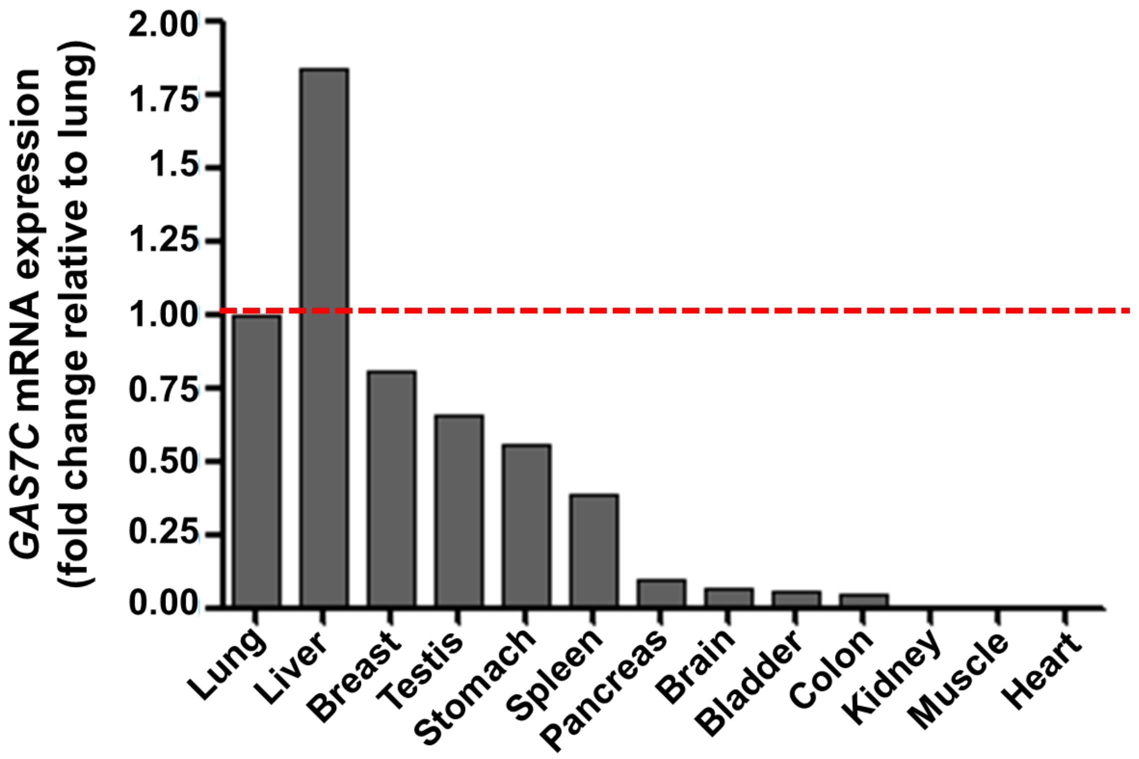

Supplementary Figure 1. The distribution of *GAS7C* mRNA in various human tissues. *GAS7C* mRNA is expressed at a high level in liver and lung tissue. The *GAS7C* mRNA expression level in lung tissue was normalized to 1.00.

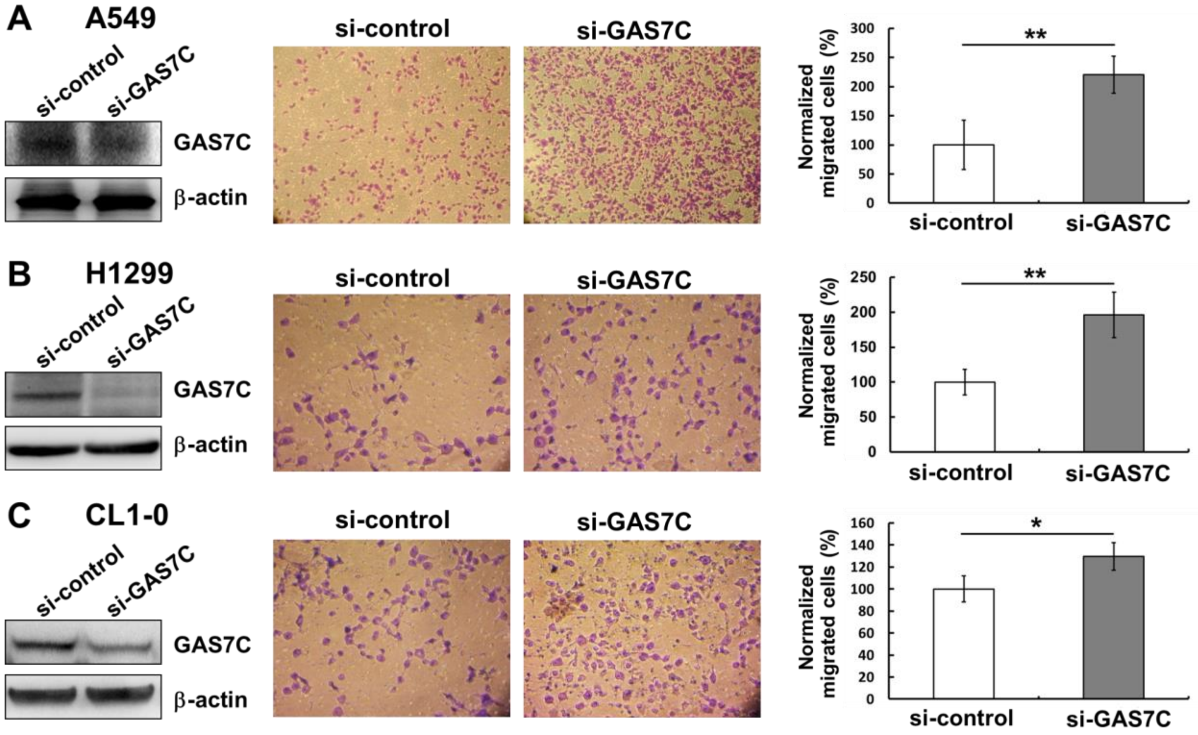

Supplementary Figure 2. Knockdown of GAS7C increases lung cancer cell migration ability. Lung cancer cells A549 **(A)**, H1299 **(B)** and CL1-0 **(C)** were transiently transfected with si-control or si-GAS7C oligos. Western blot analysis of GAS7C protein level (***left panel***), a transwell migration assay (***middle panel***) and the quantitative data (***right panel***) are shown. Data are presented as the mean ± SD from three independent experiments. * $p < 0.05$; ** $p < 0.01$.

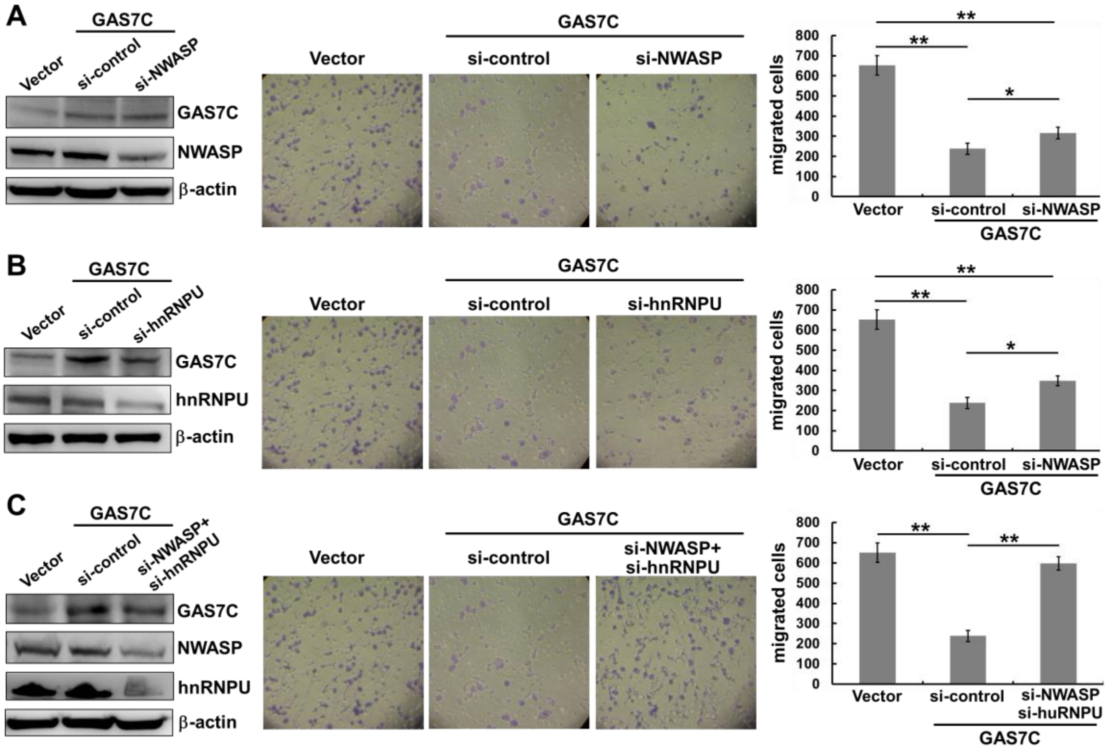

Supplementary Figure 3. The effects of GAS7C/N-WASP and GAS7C/hnRNP U on lung cancer cell motility. Knockdown of N-WASP **(A)** or hnRNP U **(B)** or both **(C)** in A549 cells overexpressing GAS7C. Double knockdown of N-WASP and hnRNP U markedly abolished the anti-migration effect of GAS7C overexpression, while the single knockdown of N-WASP or hnRNP U showed partial attenuation effect. Western blot analysis of the GAS7C protein level (***left panel***), transwell migration assay results (***middle panel***) and the quantitative data (***right panel***) are shown. Data is presented as the mean ± SD from three independent experiments. * p <0.05; ** p <0.01.

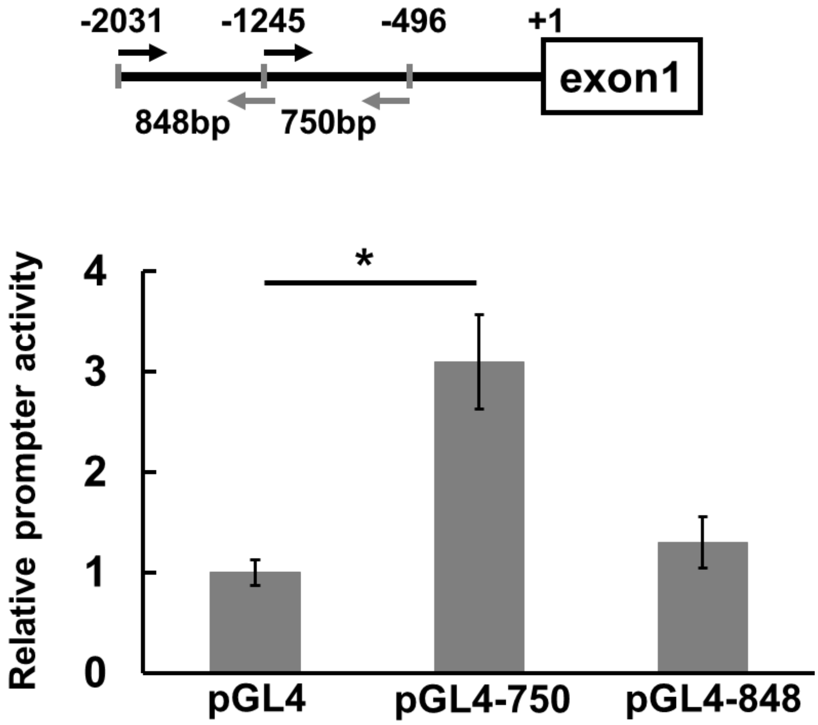

Supplementary Figure 4. Identification of the *GAS7C* promoter region. Two fragments (848 bp and 750 bp) of the *GAS7C* promoter were cloned upstream of the luciferase reporter gene (***upper***). A549 cells were co-transfected with either one of the *GAS7C* promoter construct or an internal control (Renilla luciferase vector). GAS7C activity of either the pGL-750 or pGL848 construct was compared with empty vector pGL4 (***lower***). Quantitative data are presented as the mean ± SD from three independent experiments. * $p < 0.05$.

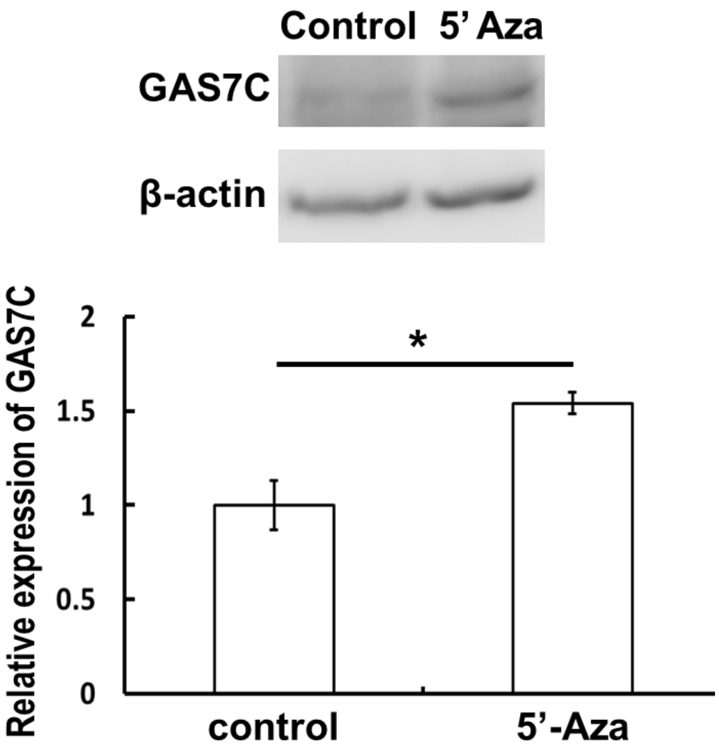

Supplementary Figure 5. mRNA and protein levels of GAS7C after DNA demethylation reagent treatment. Both protein (***upper***) and mRNA (***lower***) expression was significantly increased in the DNA demethylation reagent (Aza) treated cells compared to the DMSO treated control cells. * $p < 0.05$.

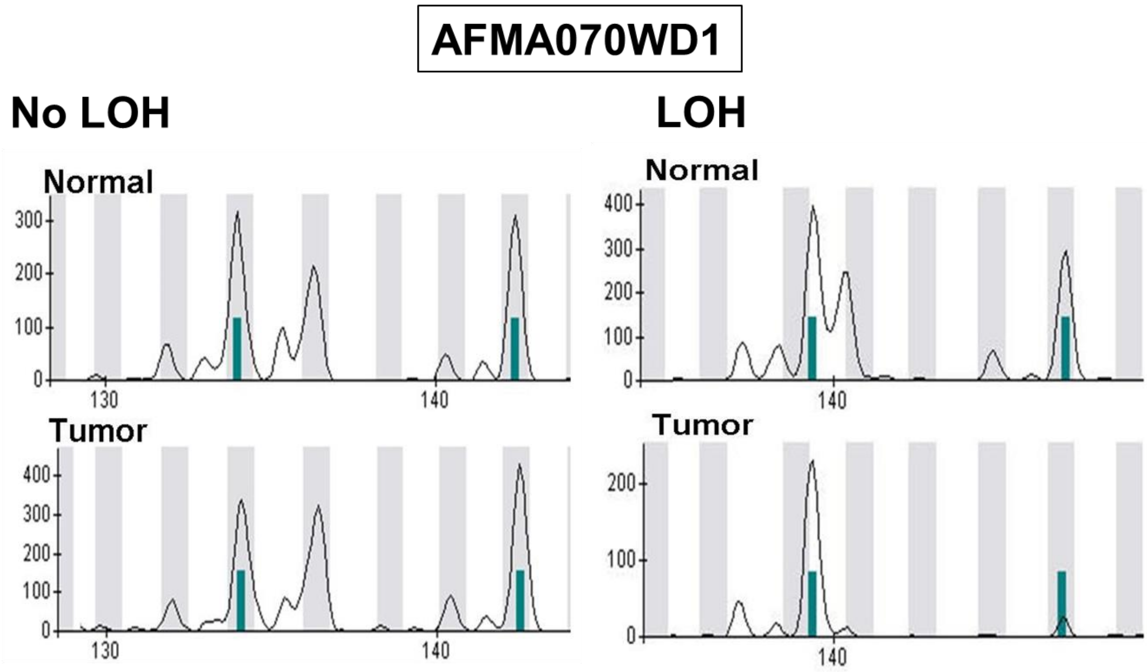

Supplementary Figure 6. The detection of LOH at the di-nucleotide repeat microsatellite marker (AFMA070WD1). The number on the X-axis indicates the length of the fragment, and the number on the Y-axis shows the fluorescence intensity of peak height (the amount of PCR product). The patient on the left did not show LOH, whereas the patient on the right did show LOH at the AFMA070WD1 microsatellite marker.

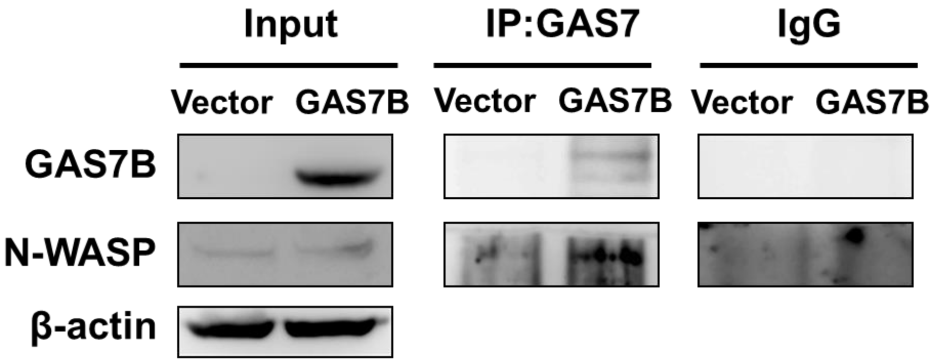

Supplementary Figure 7. GAS7B protein is associated with N-WASP. Protein lysates of CL1-0 cells expressing control (vector) or GAS7B (GAS7B) were immunoprecipitated (IP) using anti-GAS7 antibody. The IP proteins were then analyzed by Western blot using the indicated antibodies. Normal IgG served as the negative control. More N-WASP was associated with GAS7B in the GAS7B overexpressing cells than in the vector control cells.

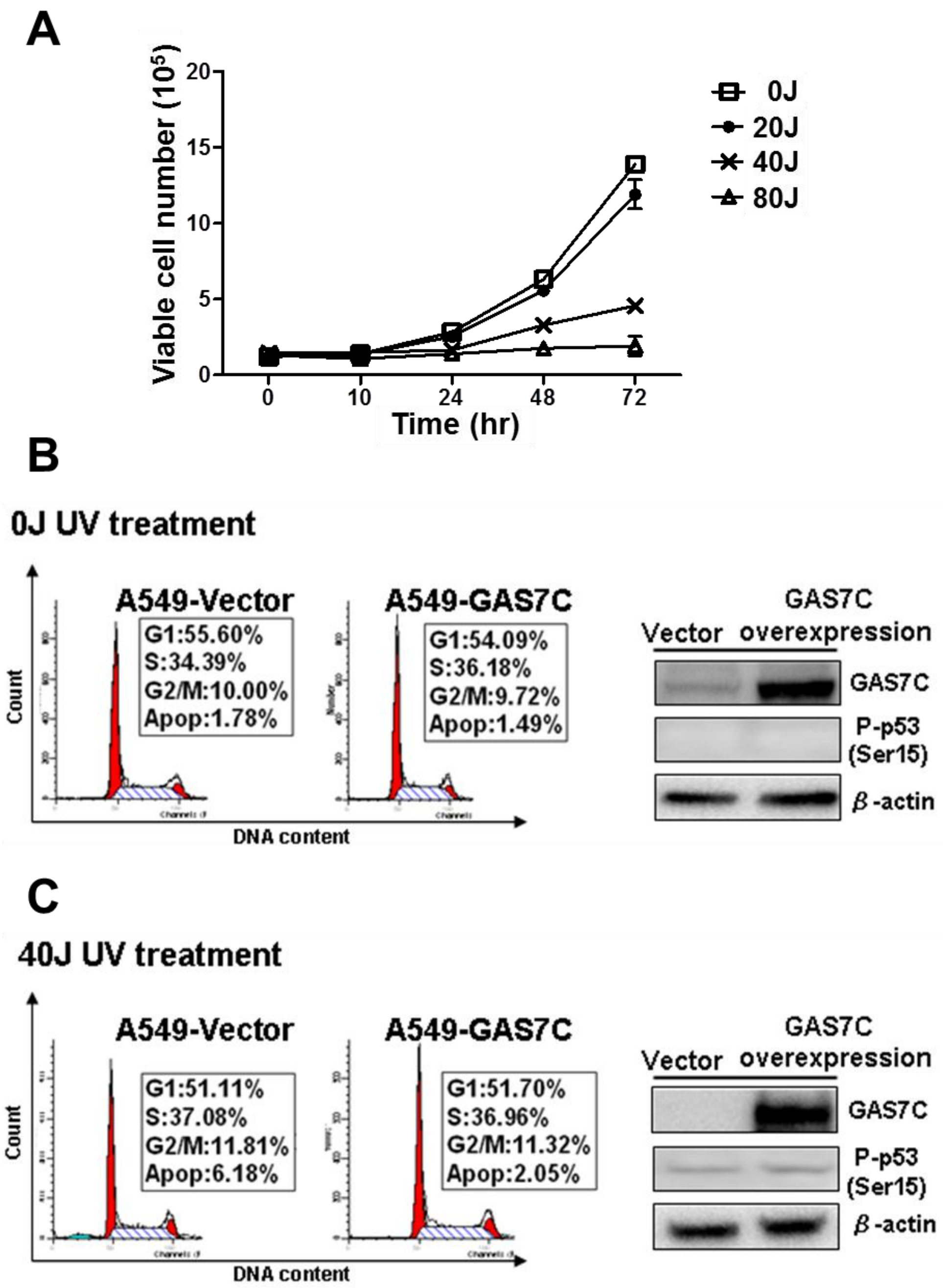

Supplementary Figure 8. The effect of GAS7C overexpression and ultraviolet (UV) treatment on cell proliferation and cell cycle distribution. (A) Proliferation rate of A549 cells following 0-80 J/m^2 UV treatment for 0-72 hr. Approximately 50% of A549 cells survived at 48 hr with 40 J/m^2 UV irradiation compared to unirradiated cells. Therefore, cells irradiated with 40 J/m^2 UV at 48 hr were used to examine the cell cycle distribution. **(B)** The

cell cycle of GAS7C expressing cells without UV treatment or **(C)** GAS7C expressing cells treated with 40 J/m^2 UV for 48 hr were analysis by FACS. The percentages of each cell cycle phase are as indicated. Western blots for GAS7C and phosphor-p53 (P-p53) are shown to indicate the protein level of GAS7C and the DNA damage response, respectively. The results indicate that GAS7C overexpression did not change the cell cycle distribution with or without a cell cycle arrest inducer, namely UV light treatment.
